# Supplementary material for: Plant diversity dynamics over space and time in a warming Arctic
Source: Nature. 2025 Apr 30;642(8068):653–61. doi: 10.1038/s41586-025-08946-8 (PMC12176628; doi:10.1038/s41586-025-08946-8)
Supplement: Supplementary file 1 — Supplementary Discussion, Methods, Supplementary Tables 1–9 and references. [file 41586_2025_8946_MOESM1_ESM.pdf]

nature portfolio

https://doi.org/10.1038/s41586-025-08946-8

Supplementary information

Plant diversity dynamics over space and time in a warming Arctic

In the format provided by the authors and unedited

Supplementary Discussion

Geography. Western North America contained the greatest number of more even communities, with a greater proportion of persisting species over time than any other regions (**Supplementary Table 3**). Eurasia experienced the greatest rates of Bray-Curtis turnover, while Greenland-Iceland had greater Jaccard turnover and more species gains than any other regions. Regional variation in biodiversity metrics might reflect landscape age following glaciation history and processes, such as the 'founder effect', where reduced genetic diversity is the result of a small number of ancestors[98], but they might also be the result of regional monitoring realities (see 'Sampling realities' below).

Richness and evenness. There was no relationship between mean plot species richness and species richness change over time. Spatial richness and evenness were correlated, with more diverse plots also being more even (**Supplementary Table 2**, **3.6**). Mean evenness (Pielou) across the Arctic was 0.7 [data bounded by 0 - 1]. Evenness was greater in more diverse plots with high forb cover and low shrub cover, and in Western North America relative to other regions (**Supplementary Table 3.6** - **10**). Overall, evenness did not change over time (**Supplementary Table 1**), but increases occurred mostly in wetter versus drier plots where forb cover increased and shrub cover decreased over time (**Supplementary Table 3.56** - **58**).

Turnover. A key difference between turnover metrics was that more species-rich plots experienced greater abundance-related turnover (Bray-Curtis), while species-poor plots had more presence-absence (Jaccard) change (**Supplementary Table 2**). We contrasted these results with a null model that yielded no significant relationship between Jaccard and richness, and a negative relationship between Bray-Curtis and richness. Thus, this contrasting pattern is likely due to less diverse plots in colder sites dominated by a few species with higher abundance that do not experience as much proportional abundance change, but whose presence-absence change will be proportionally greater. Also, more diverse plots at lower and warmer latitudes can experience more proportional abundance change potentially due to increased biotic interactions[99,100].

Temporal turnover metrics can be calculated in different ways. Here, we chose to calculate temporal turnover by comparing species composition and abundance from the start to the end time point of plot surveying (calculated as β-diversity dissimilarity metrics). This method can lead to an influence of the baseline plot composition on turnover, and does not capture changes in timeseries within the start and end time points. Calculating turnover as changes in a system state from an ordination would capture time series change, but would require data from plots that were sampled more than twice. Thus, our chosen method maximises the sample size with this dataset, given that a large number of plots were only surveyed twice (35% of all plots surveyed twice and 21.5% more than twice).

Functional group composition. Most plots were dominated (i.e., cover was > 50%) by shrubs (*n* = 1,170, 53.8%), followed by graminoid-dominated plots (689, 31.7%); plots dominated by forbs (113, 5.2%) and those where no functional groups were clearly dominant (202, 9.3%) were less common. Similarly, mean cover across plots was greater for shrubs (50%), followed by graminoids (37.4%) and forbs (12.6%, **Extended Data Figure 5a-c**). Forb, shrub and graminoid cover did not directionally change over time on average across our plots (**Supplementary Table 8**, **9**). Species-rich plots had higher forb cover and lower graminoid cover (**Supplementary Table 1**, **3.4** - **5**).

Climate change. Regional climates across the Arctic varied widely in their annual coldest quarter temperatures, but less so in their warmest quarter temperatures (MTWQ, **Extended Data Figure 4a**). All subsites experienced MTWQ increases (**Extended Data Figure 4b**), and 87.6% of subsites experienced mean annual precipitation increases over time (**Extended Data Figure 4c**). The magnitude of warming over time was greater at northern latitudes (slope = 0.00033°C/year per degree of latitude, 95% CI = 0.00018 to 0.00047, conditional and marginal R_2 = 0.009).

Sampling realities. There are a number of factors worth considering when interpreting the results in this study. First, sampling effort is uneven across the Arctic[101,102] and those data biases are represented in the ITEX+ dataset. Some regions have long-established monitoring programmes in place, while other sites are not represented partly due to the inaccessibility of some Arctic regions. Second, particular

regions might also appear as biodiversity hotspots due to the botanist effect, where sampling effort has been concentrated in one region[103]. Third, while monitoring might occur in certain regions (e.g., Russia), methods differ and are not directly comparable across the Arctic. Barriers to international collaboration between Russia and other Arctic nations can result in an increasingly biased view of Arctic change[102]. Even within the ITEX+ dataset with many sites using standardised sampling protocols[104], methods vary among sites and over time. We considered these sampling variables as fixed effects in the models in order to account for this variability (**Supplementary Table 2**).

We found that studies covering longer temporal extents had increased their abundance-related turnover (Bray-Curtis) and richness over time, but not increased species replacement (Jaccard; **Extended Data Figure 8g,h**). There was an indication that shorter studies identified more species losses, possibly suggesting that local extinctions might be due to stochastic processes or sampling effects during monitoring[105]. These two results could confirm that long-term studies tend to reflect meaningful change in communities, while shorter timescales often overrepresent real changes in Arctic communities[106]. Finally, larger plots were more even and had experienced less richness change[37,107]. Together, these findings indicate the importance of scale, local context, study design and sampling effort when drawing global inferences from local-scale monitoring.

Supplementary Methods

Data harmonisation. While preparing the ITEX+ dataset for analyses, we removed non-comparable methods like biomass and number of shoots. We removed duplicate records, i.e., those where the same plot-by-year had repeated rows for each species and abundance values (*n* = 26 plot-by-year), and retained a single copy of those records in their particular plot-by-year combination.

We converted all values to relative cover to ensure consistency across survey methods (see **Extended Data Figure 2** for a detailed account of cover conversion), with a maximum cover value of 100% within a plot. In particular for X-Y grid coordinate data, we converted multiple hits of the same species from abundance records into presence/absence at a X-Y coordinate, since this most closely resembles cover. Subsequently, we calculated the proportion of total species hits per plot and species as a function of the total number of unique species hits in a plot. We retained only live plant records and calculated cover relative to vascular plants only, removing non-vascular plants, litter, dead biomass and non-biotic hits (rock, water, etc.) given that non-vascular plants and abiotic hits were not recorded consistently across sites.

Species names were standardised following the taxonomy of The Plant List (http://www.theplantlist.org/) at the species level (i.e., subspecies or varieties were retained as species). When species were identified only to family or genus level, they were retained and converted to a morphospecies (e.g., XXXLuzula:Abisko) to reflect a unique species hit per plot. We retained only the plots with less than 10% total cover of morphospecies. Therefore, 920 plot-by-year combinations were removed (13.4% of dataset plot-by-year). Only that particular plot-by-year combination was removed, and not all the years from a given plot. Mean morphospecies cover per plot was 15.9%, thus we applied a strict filter considered to ensure site comparability with no relevant effects on our results[108].

Supplementary References

98. Stewart, L. et al. The regional species richness and genetic diversity of Arctic vegetation reflect both past glaciations and current climate. *Global Ecology and Biogeography* 25, 430–442 (2016).

99. Hillebrand, H. On the generality of the latitudinal diversity gradient. *The American Naturalist* 163, 192–211 (2004).

100. Koleff, P. & Gaston, K. J. The relationships between local and regional species richness and spatial turnover. *Global Ecology and Biogeography* 11, 363–375 (2002).

101. Metcalfe, D. B. et al. Patchy field sampling biases understanding of climate change impacts across the Arctic. *Nature Ecology and Evolution* 2, 1443–1448 (2018).

102. López-Blanco, E. et al. Towards an increasingly biased view on Arctic change. *Nature Climate Change* 14, 152–155 (2024).

103. Moerman, D. E. & Estabrook, G. F. The botanist effect: counties with maximal species richness tend to be home to universities and botanists. *Journal of Biogeography* 33, 1969–1974 (2006).

104. Henry, G. H. R. & Molau, U. Tundra plants and climate change: the International Tundra Experiment (ITEX). *Global Change Biology* 3, 1–9 (1997).

105. Matthies, D., Bräuer, I., Maibom, W. & Tscharntke, T. Population size and the risk of local extinction: empirical evidence from rare plants. *Oikos* 105, 481–488 (2004).

106. Valdez, J. W. et al. The undetectability of global biodiversity trends using local species richness. *Ecography*, e06604 (2023).

107. Drakare, S., Lennon, J. J. & Hillebrand, H. The imprint of the geographical, evolutionary and ecological context on species–area relationships. *Ecology Letters* 9, 215–227 (2006).

108. Cayuela, L., de la Cruz, M. & Ruokolainen, K. A method to incorporate the effect of taxonomic uncertainty on multivariate analyses of ecological data. *Ecography* 34, 94–102 (2011).

Supplementary Tables

Supplementary Table 1. Structure and summary results of the high-level models of richness and evenness change over time. CI are the 95% credible intervals.

Model	Model structure	Data Distribution	Sample size	Summary
Richness over time	Annual Richness ~ Year + (Year|Subsite/Plot)	Negative binomial	4,858	Slope = 0.0021, CI = -0.0002 to 0.0043
Evenness over time	Annual Evenness ~ Year + (Year|Subsite/Plot)	Beta	4,822	Slope = 0.004, CI = -0.0049 to 0.0126

Supplementary Table 2. Model summaries for spatial, two time points and temporal multivariate models, represented in the 'Group' column by the globe, hour glass and clock, respectively. These icons were commissioned for this article and designed by Alberto S. Ballesteros, who grants permission for their display here. Multiple models were fitted per response variable containing the fixed effects that affect the response variable at the same level (e.g., subsite, plot). GEO = geographical model, CLIM = climatic model, FG = functional group composition model, CHG = change over time model, PCHG = plot change over time, SUBS = subsite model. Data distributions are indicated in brackets under each response variable as follows: NB = Negative Binomial distribution, ZOIB = Zero-One-Inflated Beta distribution, ZIB = Zero-Inflated Beta distribution, Gauss. = Gaussian distribution, Temp. = Temperature, Prec. = Precipitation, Gram. = Graminoid, Chg. = Change. The 'tick mark' in the random effect column indicates where a subsite random intercepts effect has been included in the model. Dark orange circles indicate a negative significant relationship at 97.5% CI, light orange circles a negative significant relationship at 95% CI, grey circles a non-significant relationship at both 95% and 97.5% CI, light green circles a positive significant relationship at 95% CI, and dark green circles a positive significant relationship at 97.5% CI. (*) for Region and Moisture indicates a categorical variable, thus a green point indicates a difference between some of the categories and a grey circle indicates no clear difference. When models featured functional group cover or functional group change as covariates, we fitted three models, each including change in one functional group, in order to achieve convergence given that functional group proportions were inherently negatively correlated. These three models included all the same covariates except for the functional group in question and are all represented in the same row under FG and PCHG models. Here, a pie chart showcases slopes for the three different functional group models per response variable, and the initial indicates the functional group that is different (S = shrub, G = graminoid, F = forb). The symbol '*' indicates that richness was significant in the graminoid model (97.5%) and in the forb model (95%), but non-significant in the shrub model (97.5%). For temporal models, change over time was modelled for each plot and the slopes from those linear regressions are the response variables used in subsequent models. For Jaccard and Bray-Curtis, the change in PCoA values per plot was calculated through Cartesian coordinates, and also as the difference in distance to centroid for each plot, and those values were the response variables in the subsequent models.

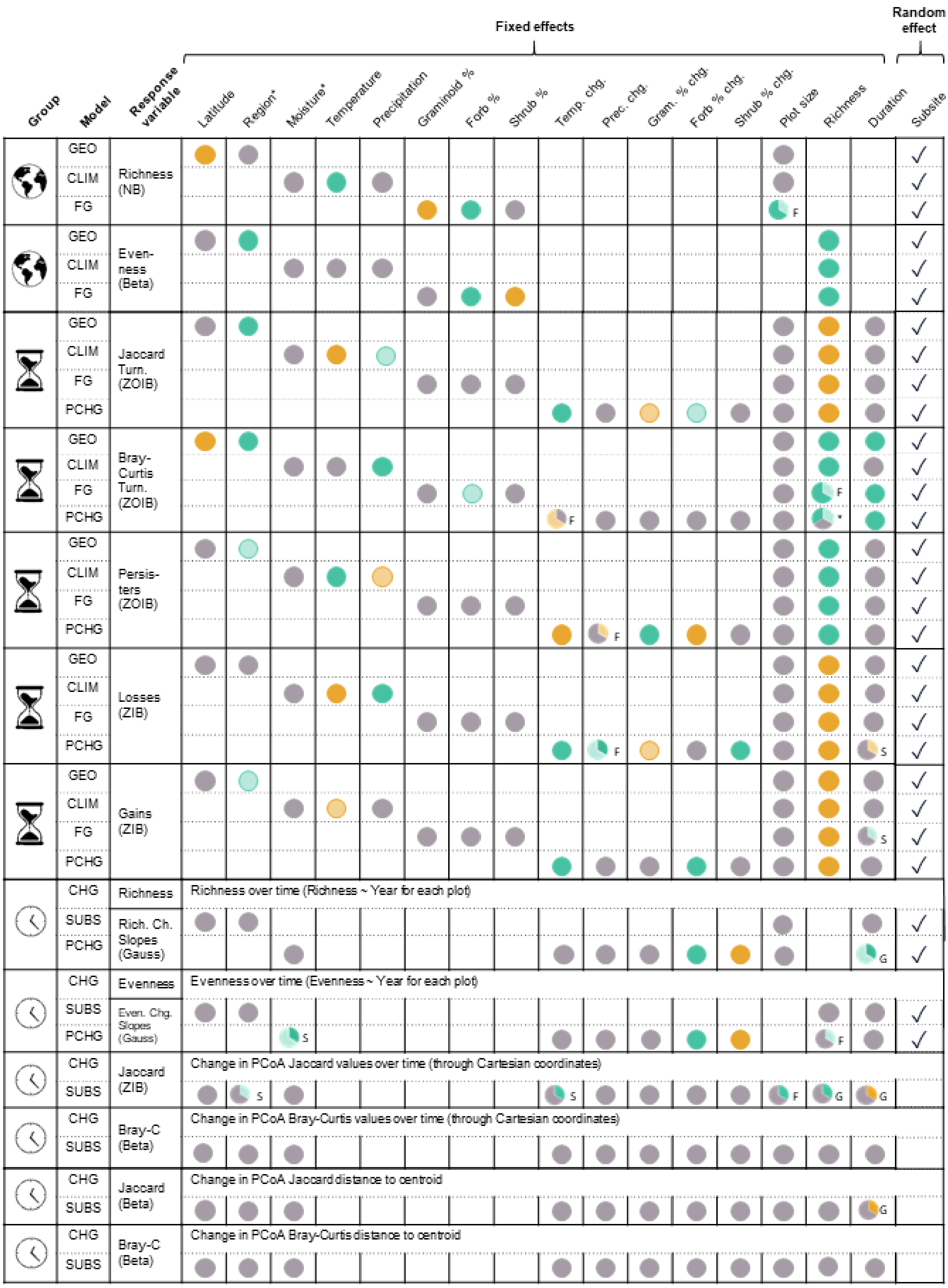
Fixed effects
Random effect
Group
Model
Response variable
Latitude
Region*
Moisture*
Temperature
Precipitation
Graminoid %
Forb %
Shrub %
Temp. chg.
Prec. chg.
Gram. % chg.
Forb % chg.
Shrub % chg.
Plot size
Richness
Duration
Subsite
GEO
CLIM
FG
PCHG
CHG
SUBS
Richness (NB)
Even-ness (Beta)
Jaccard Turn. (ZOIB)
Bray-Curtis Turn. (ZOIB)
Persis-ters (ZOIB)
Losses (ZIB)
Gains (ZIB)
Richness
Rich. Ch. Slopes (Gauss)
Richness over time (Richness ~ Year for each plot)
Evenness
Even. Chg. Slopes (Gauss)
Evenness over time (Evenness ~ Year for each plot)
Jaccard (ZIB)
Change in PCoA Jaccard values over time (through Cartesian coordinates)
Bray-C (Beta)
Change in PCoA Bray-Curtis values over time (through Cartesian coordinates)
Jaccard (Beta)
Change in PCoA Jaccard distance to centroid
Bray-C (Beta)
Change in PCoA Bray-Curtis distance to centroid

Supplementary Table 3. Summary of the key Bayesian models fitted in this study (as shown in **Supplementary Table 2**). Model outputs are shown for spatial, two time-point and temporal models. CI means credible intervals. Parameters in bold show those predictor variables whose 95% CI do not overlap zero (except for categorical variables), and thus can be considered to have a 'significant' effect in the response variable.

Model number	Model name	Term	Estimate	Std. error	Lower 95% CI	Upper 95% CI
**Spatial models**						
**1**	Richness GEO	b_Intercept	3.942	0.556	2.856	5.034
		**b_LAT**	**-0.029**	**0.008**	**-0.045**	**-0.013**
		b_LogPlotSize	0.027	0.053	-0.077	0.131
		b_RegionGreenIceLand	0.006	0.101	-0.19	0.206
		b_RegionNorthAmericaMEast	-0.057	0.133	-0.323	0.199
		b_RegionNorthAmericaMWest	0.106	0.11	-0.105	0.325
		sd_SiteSubsite__Intercept	0.437	0.029	0.384	0.497
		shape	603.93	176.896	326.976	1019.587
		lprior	-20.145	2.05	-24.861	-16.809
**2**	Richness CLIM	b_Intercept	1.332	0.134	1.063	1.596
		**b_warmq**	**0.062**	**0.016**	**0.031**	**0.093**
		b_prec	0.00009	0.00014	-0.0002	0.0004
		b_MOISTUREMOIST	0.204	0.103	-0.001	0.402
		b_MOISTUREWET	0.1	0.125	-0.147	0.338
		b_LogPlotSize	-0.024	0.068	-0.159	0.11
		sd_SiteSubsite__Intercept	0.405	0.031	0.348	0.47
		shape	555.492	169.364	295.487	953.224
		lprior	-19.572	1.984	-24.128	-16.391
**3**	Richness FG - Shrub	b_Intercept	1.864	0.078	1.713	2.014
		b_ShrubDivided	0.053	0.073	-0.09	0.195
		**b_LogPlotSize**	**0.164**	**0.072**	**0.023**	**0.304**
		sd_SiteSubsite__Intercept	0.465	0.044	0.386	0.562
		shape	422.404	147.341	208.154	765.103
		lprior	-17.964	1.802	-22.034	-15.176
**4**	Richness FG - Forb	b_Intercept	1.788	0.072	1.645	1.93
		**b_ForbDivided**	**0.974**	**0.124**	**0.725**	**1.219**
		**b_LogPlotSize**	**0.153**	**0.07**	**0.01**	**0.289**
		sd_SiteSubsite__Intercept	0.452	0.042	0.378	0.543
		shape	444.089	153.933	218.58	810.127
		lprior	-18.229	1.866	-22.538	-15.328
**5**	Richness FG - Graminoid	b_Intercept	2.041	0.079	1.885	2.194
		**b_GramDivided**	**-0.37**	**0.074**	**-0.513**	**-0.227**
		**b_LogPlotSize**	**0.186**	**0.072**	**0.043**	**0.327**
		sd_SiteSubsite__Intercept	0.475	0.045	0.395	0.574
		shape	433.55	153.022	212.93	797.25
		lprior	-18.102	1.86	-22.4	-15.249
**6**	Evenness GEO	b_Intercept	-0.847	0.678	-2.176	0.480
		b_LAT	0.017	0.01	-0.002	0.035
		b_RegionGreenIceLand	-0.062	0.113	-0.283	0.156
		b_RegionNorthAmericaMEast	0.074	0.163	-0.250	0.381
		b_RegionNorthAmericaMWest	0.449	0.124	0.202	0.696
		**b_MeanRichness**	**0.046**	**0.007**	**0.031**	**0.060**
		sd_SiteSubsite__Intercept	0.502	0.036	0.437	0.577
		phi	10.373	0.323	9.750	11.002

		lprior	-10.296	0.036	-10.364	-10.225
**7**	Evenness CLIM	b_Intercept	0.476	0.189	0.101	0.837
		b_MOISTUREMOIST	0.010	0.13	-0.24	0.267
		b_MOISTUREWET	0.091	0.175	-0.248	0.431
		b_warmq	-0.024	0.022	-0.07	0.02
		b_prec	-0.00012	0.0002	-0.04	0.075
		**b_MeanRichness**	**0.06**	**0.008**	**0.044**	**0.076**
		sd_SiteSubsite__Intercept	0.576	0.044	0.496	0.670
		phi	12.191	0.435	11.37	13.072
		lprior	-10.474	0.041	-10.556	-10.393
**8**	Evenness FG - Shrub	b_Intercept	0.423	0.096	0.237	0.615
		**b_ShrubDivided**	**-0.294**	**0.090**	**-0.468**	**-0.118**
		**b_MeanRichness**	**0.056**	**0.008**	**0.041**	**0.072**
		sd_SiteSubsite__Intercept	0.587	0.044	0.508	0.680
		phi	12.303	0.426	11.466	13.135
		lprior	-10.486	0.040	-10.564	-10.406
**9**	Evenness FG - Forb	b_Intercept	0.252	0.082	0.093	0.408
		**b_ForbDivided**	**1.177**	**0.16**	**0.868**	**1.487**
		**b_MeanRichness**	**0.042**	**0.008**	**0.026**	**0.058**
		sd_SiteSubsite__Intercept	0.579	0.043	0.503	0.670
		phi	12.63	0.444	11.775	13.530
		lprior	-10.513	0.04	-10.594	-10.433
**10**	Evenness FG - Graminoid	b_Intercept	0.313	0.092	0.134	0.493
		b_GramDivided	-0.092	0.094	-0.277	0.089
		**b_MeanRichness**	**0.057**	**0.008**	**0.042**	**0.072**
		sd_SiteSubsite__Intercept	0.575	0.043	0.498	0.663
		phi	12.191	0.412	11.389	12.991
		lprior	-10.474	0.039	-10.551	-10.395
**Two time-point models**						
**11**	Jaccard GEO	b_Intercept	-0.706	0.734	-2.123	0.753
		b_zoi_Intercept	-0.338	0.056	-0.45	-0.228
		b_coi_Intercept	-5.838	0.807	-7.706	-4.557
		b_LAT	0.011	0.01	-0.009	0.031
		b_RegionGreenIceLand	0.421	0.153	0.115	0.718
		b_RegionNorthAmericaMEast	-0.114	0.174	-0.452	0.224
		b_RegionNorthAmericaMWest	-0.099	0.099	-0.292	0.099
		b_LogPlotSize	0.06	0.099	-0.129	0.253
		**b_MeanRichness**	**-0.059**	**0.01**	**-0.08**	**-0.041**
		b_duration	-0.005	0.007	-0.018	0.008
		sd_SiteSubsite__Intercept	0.299	0.041	0.226	0.383
		phi	16.573	0.866	14.927	18.337
		lprior	-18.027	0.805	-19.886	-16.749
**12**	Jaccard CLIM	b_Intercept	0.266	0.215	-0.153	0.691
		b_zoi_Intercept	-0.335	0.057	-0.448	-0.224
		b_coi_Intercept	-5.804	0.818	-7.621	-4.516
		b_MOISTUREMOIST	-0.056	0.111	-0.278	0.163
		b_MOISTUREWET	0.113	0.147	-0.179	0.411
		**b_warmq**	**-0.055**	**0.02**	**-0.093**	**-0.016**
		**b_prec**	**0.001**	**0.0002**	**0.0004**	**0.01**
		b_duration	-0.005	0.007	-0.019	0.009
		b_LogPlotSize	0.065	0.127	-0.189	0.316
		**b_MeanRichness**	**-0.062**	**0.01**	**-0.082**	**-0.043**

		sd_SiteSubsite__Intercept	0.307	0.042	0.231	0.397
		phi	16.734	0.912	15.001	18.635
		lprior	-18.005	0.817	-19.826	-16.719
**13**	Jaccard FG - Shrub	b_Intercept	0.038	0.156	-0.266	0.347
		b_zoi_Intercept	-0.338	0.056	-0.448	-0.225
		b_coi_Intercept	-5.854	0.82	-7.69	-4.522
		b_ShrubDivided	-0.11	0.105	-0.319	0.101
		b_LogPlotSize	-0.068	0.09	-0.24	0.11
		**b_MeanRichness**	**-0.068**	**0.01**	**-0.088**	**-0.048**
		b_duration	0.002	0.006	-0.011	0.014
		sd_SiteSubsite__Intercept	0.32	0.041	0.246	0.409
		phi	16.591	0.928	14.81	18.458
		lprior	-18.043	0.816	-19.854	-16.724
**14**	Jaccard FG - Forb	b_Intercept	-0.02	0.139	-0.287	0.259
		b_zoi_Intercept	-0.338	0.056	-0.445	-0.23
		b_coi_Intercept	-5.836	0.785	-7.594	-4.537
		b_ForbDivided	0.128	0.234	-0.332	0.58
		b_LogPlotSize	-0.064	0.09	-0.238	0.117
		**b_MeanRichness**	**-0.068**	**0.01**	**-0.09**	**-0.049**
		b_duration	0.001	0.006	-0.012	0.013
		sd_SiteSubsite__Intercept	0.322	0.04	0.248	0.406
		phi	16.601	0.907	14.902	18.41
		lprior	-18.026	0.783	-19.805	-16.745
**15**	Jaccard FG - Graminoid	b_Intercept	-0.067	0.146	-0.356	0.226
		b_zoi_Intercept	-0.338	0.057	-0.451	-0.227
		b_coi_Intercept	-5.848	0.795	-7.664	-4.567
		b_GramDivided	0.101	0.114	-0.123	0.328
		b_LogPlotSize	-0.068	0.091	-0.238	0.115
		**b_MeanRichness**	**-0.067**	**0.01**	**-0.085**	**-0.048**
		b_duration	0.002	0.006	-0.011	0.014
		sd_SiteSubsite__Intercept	0.322	0.041	0.248	0.408
		phi	16.594	0.907	14.864	18.404
		lprior	-18.038	0.794	-19.832	-16.748
**16**	Jaccard PCHG - Shrub	b_Intercept	-0.48	0.189	-0.848	-0.106
		b_zoi_Intercept	-0.456	0.062	-0.577	-0.338
		b_coi_Intercept	-5.629	0.823	-7.551	-4.286
		**b_WarmQSlope**	**14.455**	**3.928**	**6.86**	**22.402**
		b_PrecSlope	0.023	0.013	-0.002	0.049
		b_ShrubSlope	0.009	0.015	-0.02	0.039
		b_LogPlotSize	0.014	0.093	-0.165	0.203
		**b_MeanRichness**	**-0.069**	**0.01**	**-0.089**	**-0.049**
		b_duration	-0.005	0.007	-0.018	0.009
		sd_SiteSubsite__Intercept	0.311	0.041	0.236	0.395
		phi	16.695	0.952	14.897	18.554
		lprior	-17.856	0.82	-19.799	-16.517
**17**	Jaccard PCHG - Forb	b_Intercept	-0.516	0.196	-0.896	-0.135
		b_zoi_Intercept	-0.646	0.067	-0.779	-0.512
		b_coi_Intercept	-5.41	0.803	-7.269	-4.125
		**b_WarmQSlope**	**13.645**	**4.038**	**5.763**	**21.597**
		b_PrecSlope	0.022	0.013	-0.004	0.048
		b_ForbSlope	0.039	0.021	-0.002	0.08
		b_LogPlotSize	0.005	0.095	-0.184	0.194

		**b_MeanRichness**	**-0.064**	**0.01**	**-0.085**	**-0.044**
		b_duration	-0.002	0.007	-0.016	0.01
		sd_SiteSubsite__Intercept	0.304	0.041	0.23	0.388
		phi	15.337	0.873	13.703	17.08
		lprior	-17.592	0.799	-19.444	-16.319
**18**	Jaccard PCHG - Graminoid	b_Intercept	-0.506	0.178	-0.85	-0.143
		b_zoi_Intercept	-0.369	0.058	-0.482	-0.259
		b_coi_Intercept	-6.8	1.243	-9.82	-4.943
		**b_WarmQSlope**	**13.873**	**3.719**	**6.489**	**21.337**
		b_PrecSlope	0.02	0.013	-0.005	0.044
		**b_GraminoidSlope**	**-0.033**	**0.015**	**-0.063**	**-0.003**
		b_LogPlotSize	-0.015	0.09	-0.184	0.167
		**b_MeanRichness**	**-0.067**	**0.01**	**-0.086**	**-0.048**
		b_duration	-0.003	0.006	-0.015	0.01
		sd_SiteSubsite__Intercept	0.295	0.038	0.226	0.375
		phi	16.738	0.881	15.06	18.509
		lprior	-19.006	1.241	-22.014	-17.157
**19**	Bray-Curtis GEO	b_Intercept	1.35	0.989	-0.628	3.247
		b_zoi_Intercept	-5.123	0.368	-5.911	-4.468
		b_coi_Intercept	-1.452	0.88	-3.354	0.085
		**b_LAT**	**-0.033**	**0.014**	**-0.06**	**-0.005**
		b_RegionGreenIceLand	-0.459	0.196	-0.828	-0.07
		b_RegionNorthAmericaMEast	-0.313	0.229	-0.766	0.146
		b_RegionNorthAmericaMWest	-0.458	0.133	-0.721	-0.198
		b_LogPlotSize	-0.014	0.118	-0.24	0.222
		**b_MeanRichness**	**0.027**	**0.01**	**0.007**	**0.047**
		**b_duration**	**0.023**	**0.008**	**0.008**	**0.04**
		sd_SiteSubsite__Intercept	0.445	0.046	0.362	0.542
		phi	8.828	0.349	8.162	9.518
		lprior	-17.223	0.674	-18.806	-16.213
**20**	Bray-Curtis CLIM	b_Intercept	-1.253	0.223	-1.696	-0.811
		b_zoi_Intercept	-5.099	0.375	-5.924	-4.431
		b_coi_Intercept	-1.444	0.89	-3.386	0.109
		b_MOISTUREMOIST	-0.22	0.135	-0.482	0.05
		b_MOISTUREWET	0.072	0.177	-0.264	0.424
		b_warmq	-0.025	0.022	-0.069	0.019
		**b_prec**	**0.001**	**0.0003**	**0.001**	**0.002**
		b_duration	0.014	0.008	-0.001	0.03
		b_LogPlotSize	0.042	0.138	-0.237	0.308
		**b_MeanRichness**	**0.032**	**0.01**	**0.012**	**0.052**
		sd_SiteSubsite__Intercept	0.407	0.048	0.323	0.509
		phi	8.735	0.353	8.069	9.439
		lprior	-17.177	0.677	-18.792	-16.145
**21**	Bray-Curtis FG - Shrub	b_Intercept	-1.127	0.17	-1.46	-0.79
		b_zoi_Intercept	-5.119	0.363	-5.892	-4.471
		b_coi_Intercept	-1.447	0.896	-3.416	0.11
		b_ShrubDivided	-0.198	0.122	-0.435	0.04
		b_LogPlotSize	0.008	0.119	-0.219	0.244
		**b_MeanRichness**	**0.028**	**0.01**	**0.008**	**0.048**
		**b_duration**	**0.026**	**0.008**	**0.01**	**0.041**
		sd_SiteSubsite__Intercept	0.522	0.049	0.435	0.626
		phi	8.836	0.349	8.166	9.529

		lprior	-17.222	0.675	-18.845	-16.203
**22**	Bray-Curtis FG - Forb	b_Intercept	-1.232	0.157	-1.538	-0.926
		b_zoi_Intercept	-5.121	0.36	-5.903	-4.467
		b_coi_Intercept	-1.465	0.895	-3.474	0.076
		**b_ForbDivided**	**0.528**	**0.261**	**0.009**	**1.033**
		b_LogPlotSize	0.014	0.123	-0.226	0.259
		**b_MeanRichness**	**0.022**	**0.011**	**0.00003**	**0.044**
		**b_duration**	**0.025**	**0.008**	**0.009**	**0.04**
		sd_SiteSubsite__Intercept	0.521	0.049	0.432	0.628
		phi	8.845	0.354	8.153	9.545
		lprior	-17.235	0.679	-18.904	-16.243
**23**	Bray-Curtis FG - Graminoid	b_Intercept	-1.287	0.168	-1.62	-0.967
		b_zoi_Intercept	-5.115	0.358	-5.878	-4.469
		b_coi_Intercept	-1.469	0.907	-3.472	0.122
		b_GramDivided	0.097	0.13	-0.159	0.35
		b_LogPlotSize	0.004	0.122	-0.232	0.244
		**b_MeanRichness**	**0.03**	**0.01**	**0.009**	**0.051**
		**b_duration**	**0.025**	**0.008**	**0.01**	**0.041**
		sd_SiteSubsite__Intercept	0.524	0.049	0.436	0.626
		phi	8.825	0.35	8.153	9.501
		lprior	-17.234	0.691	-18.896	-16.216
**24**	Bray-Curtis PCHG - Shrub	b_Intercept	-0.761	0.261	-1.283	-0.256
		b_zoi_Intercept	-5.478	0.48	-6.51	-4.647
		b_coi_Intercept	-0.849	0.981	-2.907	0.96
		**b_WarmQSlope**	**-13.174**	**5.867**	**-24.276**	**-1.717**
		b_PrecSlope	0.004	0.018	-0.031	0.04
		b_ShrubSlope	-0.03	0.016	-0.063	0.001
		b_LogPlotSize	0.034	0.127	-0.215	0.276
		b_MeanRichness	0.022	0.012	-0.002	0.044
		**b_duration**	**0.027**	**0.009**	**0.009**	**0.044**
		sd_SiteSubsite__Intercept	0.511	0.051	0.419	0.617
		phi	8.865	0.376	8.145	9.614
		lprior	-17.34	0.672	-18.941	-16.3
**25**	Bray-Curtis PCHG - Forb	b_Intercept	-0.925	0.285	-1.487	-0.365
		b_zoi_Intercept	-5.642	0.531	-6.796	-4.734
		b_coi_Intercept	-0.507	1.009	-2.562	1.393
		b_WarmQSlope	-9.214	6.605	-22.312	3.64
		b_PrecSlope	0.012	0.019	-0.026	0.049
		b_ForbSlope	0.04	0.02	-0.0003	0.08
		b_LogPlotSize	0.031	0.134	-0.232	0.289
		**b_MeanRichness**	**0.024**	**0.011**	**0.003**	**0.045**
		**b_duration**	**0.024**	**0.008**	**0.007**	**0.04**
		sd_SiteSubsite__Intercept	0.564	0.057	0.464	0.688
		phi	11.341	0.497	10.398	12.351
		lprior	-17.704	0.653	-19.191	-16.653
**26**	Bray-Curtis PCHG - Graminoid	b_Intercept	-0.786	0.253	-1.288	-0.291
		b_zoi_Intercept	-5.62	0.476	-6.67	-4.793
		b_coi_Intercept	-2.104	1.39	-5.338	0.065
		**b_WarmQSlope**	**-12.887**	**5.92**	**-24.604**	**-1.144**
		b_PrecSlope	-0.004	0.018	-0.038	0.032
		b_GraminoidSlope	0.013	0.017	-0.02	0.045
		b_LogPlotSize	-0.031	0.13	-0.289	0.219

		**b_MeanRichness**	**0.026**	**0.011**	**0.004**	**0.047**
		**b_duration**	**0.027**	**0.008**	**0.012**	**0.043**
		sd_SiteSubsite__Intercept	0.52	0.051	0.429	0.626
		phi	8.802	0.35	8.145	9.518
		lprior	-18.228	1.197	-21.224	-16.621
**27**	Persisters GEO	b_Intercept	1.629	0.831	-0.034	3.205
		b_zoi_Intercept	-2.388	0.101	-2.589	-2.192
		b_coi_Intercept	4.221	0.788	2.912	6.008
		b_LAT	-0.02	0.012	-0.043	0.004
		b_RegionGreenIceLand	-0.408	0.168	-0.74	-0.077
		b_RegionNorthAmericaMEast	0.11	0.194	-0.277	0.485
		b_RegionNorthAmericaMWest	0.171	0.113	-0.053	0.397
		b_LogPlotSize	-0.158	0.106	-0.363	0.048
		**b_MeanRichness**	**0.025**	**0.01**	**0.006**	**0.045**
		b_Duration	-0.005	0.007	-0.019	0.009
		sd_SiteSubsite__Intercept	0.37	0.042	0.293	0.459
		phi	10.665	0.439	9.817	11.535
		lprior	-17.093	0.772	-18.872	-15.829
**28**	Persisters CLIM	b_Intercept	-0.06	0.206	-0.458	0.345
		b_zoi_Intercept	-2.364	0.102	-2.566	-2.17
		b_coi_Intercept	4.242	0.807	2.916	6.062
		b_MOISTUREMOIST	0.059	0.122	-0.174	0.306
		b_MOISTUREWET	-0.012	0.165	-0.333	0.318
		**b_warmq**	**0.074**	**0.021**	**0.032**	**0.114**
		**b_prec**	**-0.001**	**0.0003**	**-0.001**	**-0.00001**
		b_Duration	-0.007	0.007	-0.022	0.007
		b_LogPlotSize	-0.066	0.134	-0.335	0.2
		**b_MeanRichness**	**0.026**	**0.01**	**0.007**	**0.046**
		sd_SiteSubsite__Intercept	0.375	0.045	0.293	0.47
		phi	10.869	0.462	9.973	11.797
		lprior	-17.114	0.79	-18.936	-15.843
**29**	Persisters FG - Shrub	b_Intercept	0.215	0.152	-0.081	0.512
		b_zoi_Intercept	-2.39	0.101	-2.588	-2.198
		b_coi_Intercept	4.231	0.786	2.966	6.013
		b_ShrubDivided	0.116	0.109	-0.104	0.323
		b_LogPlotSize	-0.025	0.102	-0.227	0.177
		**b_MeanRichness**	**0.035**	**0.009**	**0.016**	**0.053**
		b_Duration	-0.011	0.007	-0.024	0.003
		sd_SiteSubsite__Intercept	0.405	0.043	0.327	0.497
		phi	10.679	0.435	9.851	11.565
		lprior	-17.105	0.772	-18.867	-15.861
**30**	Persisters FG - Forb	b_Intercept	0.297	0.141	0.013	0.57
		b_zoi_Intercept	-2.389	0.101	-2.59	-2.195
		b_coi_Intercept	4.232	0.82	2.919	6.154
		b_ForbDivided	-0.262	0.243	-0.727	0.223
		b_LogPlotSize	-0.023	0.101	-0.221	0.176
		**b_MeanRichness**	**0.036**	**0.01**	**0.017**	**0.056**
		b_Duration	-0.011	0.007	-0.024	0.003
		sd_SiteSubsite__Intercept	0.401	0.044	0.322	0.495
		phi	10.672	0.453	9.829	11.588
		lprior	-17.105	0.802	-19	-15.834
**31**		b_Intercept	0.324	0.145	0.031	0.608

	Persisters FG - Graminoid	b_zoi_Intercept	-2.389	0.1	-2.591	-2.198
		b_coi_Intercept	4.235	0.79	2.958	6.071
		b_GramDivided	-0.07	0.112	-0.29	0.156
		b_LogPlotSize	-0.021	0.101	-0.218	0.173
		**b_MeanRichness**	**0.033**	**0.009**	**0.015**	**0.052**
		b_Duration	-0.011	0.007	-0.024	0.002
		sd_SiteSubsite__Intercept	0.407	0.044	0.33	0.502
		phi	10.676	0.444	9.831	11.562
		lprior	-17.108	0.772	-18.9	-15.89
**32**	Persisters PCHG - Shrub	b_Intercept	0.903	0.207	0.503	1.306
		b_zoi_Intercept	-2.658	0.123	-2.904	-2.423
		b_coi_Intercept	3.806	0.785	2.505	5.567
		**b_WarmQSlope**	**-18.053**	**4.465**	**-27.038**	**-9.45**
		b_PrecSlope	-0.022	0.014	-0.05	0.006
		b_ShrubSlope	-0.023	0.014	-0.05	0.005
		b_LogPlotSize	-0.087	0.103	-0.292	0.119
		**b_MeanRichness**	**0.029**	**0.01**	**0.008**	**0.049**
		b_Duration	-0.003	0.007	-0.017	0.011
		sd_SiteSubsite__Intercept	0.38	0.043	0.303	0.475
		phi	10.795	0.485	9.869	11.786
		lprior	-16.941	0.759	-18.624	-15.718
**33**	Persisters PCHG - Forb	b_Intercept	0.776	0.223	0.338	1.204
		b_zoi_Intercept	-3.135	0.159	-3.454	-2.832
		b_coi_Intercept	3.252	0.79	1.946	4.979
		**b_WarmQSlope**	**-17.508**	**4.875**	**-27.091**	**-7.987**
		**b_PrecSlope**	**-0.033**	**0.015**	**-0.063**	**-0.003**
		**b_ForbSlope**	**-0.046**	**0.02**	**-0.085**	**-0.008**
		b_LogPlotSize	-0.156	0.107	-0.367	0.053
		**b_MeanRichness**	**0.037**	**0.01**	**0.018**	**0.057**
		b_Duration	-0.003	0.007	-0.017	0.011
		sd_SiteSubsite__Intercept	0.398	0.045	0.317	0.493
		phi	11.113	0.508	10.134	12.123
		lprior	-16.886	0.752	-18.542	-15.674
**34**	Persisters PCHG - Graminoid	b_Intercept	0.908	0.193	0.518	1.285
		b_zoi_Intercept	-2.461	0.105	-2.667	-2.261
		b_coi_Intercept	5.164	1.283	3.292	8.244
		**b_WarmQSlope**	**-19.262**	**4.25**	**-27.615**	**-10.838**
		b_PrecSlope	-0.022	0.014	-0.049	0.004
		**b_GraminoidSlope**	**0.048**	**0.015**	**0.019**	**0.077**
		b_LogPlotSize	-0.104	0.098	-0.3	0.082
		**b_MeanRichness**	**0.037**	**0.01**	**0.017**	**0.056**
		b_Duration	-0.006	0.007	-0.018	0.008
		sd_SiteSubsite__Intercept	0.364	0.04	0.292	0.447
		phi	10.889	0.462	9.979	11.811
		lprior	-18.104	1.272	-21.14	-16.253
**35**	Losses GEO	b_Intercept	-1.271	0.856	-2.974	0.392
		b_zi_Intercept	-0.942	0.062	-1.066	-0.82
		b_LAT	0.013	0.012	-0.01	0.037
		b_RegionGreenIceLand	0.101	0.186	-0.276	0.457
		b_RegionNorthAmericaMEast	-0.066	0.2	-0.467	0.32
		b_RegionNorthAmericaMWest	-0.191	0.115	-0.412	0.04
		b_LogPlotSize	0.159	0.111	-0.058	0.387

		**b_MeanRichness**	**-0.067**	**0.011**	**-0.089**	**-0.046**
		b_Duration	-0.012	0.007	-0.026	0.003
		sd_SiteSubsite__Intercept	0.384	0.046	0.3	0.48
		phi	15.739	0.766	14.268	17.326
		lprior	-12.425	0.065	-12.552	-12.297
**36**	Losses CLIM	b_Intercept	-0.114	0.197	-0.495	0.275
		b_zi_Intercept	-0.941	0.064	-1.066	-0.814
		b_MOISTUREMOIST	-0.067	0.118	-0.295	0.173
		b_MOISTUREWET	0.005	0.161	-0.309	0.319
		**b_warmq**	**-0.069**	**0.019**	**-0.107**	**-0.03**
		**b_prec**	**0.001**	**0.0003**	**0.0001**	**0.001**
		b_Duration	-0.014	0.007	-0.028	0.001
		b_LogPlotSize	0.215	0.13	-0.042	0.475
		**b_MeanRichness**	**-0.062**	**0.011**	**-0.083**	**-0.041**
		sd_SiteSubsite__Intercept	0.338	0.046	0.255	0.433
		phi	15.85	0.773	14.367	17.391
		lprior	-12.426	0.065	-12.553	-12.299
**37**	Losses FG - Shrub	b_Intercept	-0.345	0.157	-0.653	-0.036
		b_zi_Intercept	-0.941	0.063	-1.065	-0.819
		b_ShrubDivided	-0.142	0.111	-0.351	0.076
		b_LogPlotSize	0.096	0.101	-0.092	0.302
		**b_MeanRichness**	**-0.074**	**0.011**	**-0.094**	**-0.053**
		b_Duration	-0.008	0.007	-0.022	0.006
		sd_SiteSubsite__Intercept	0.388	0.045	0.309	0.484
		phi	15.746	0.767	14.284	17.291
		lprior	-12.416	0.065	-12.544	-12.289
**38**	Losses FG - Forb	b_Intercept	-0.432	0.144	-0.713	-0.148
		b_zi_Intercept	-0.941	0.062	-1.063	-0.819
		b_ForbDivided	0.046	0.244	-0.431	0.517
		b_LogPlotSize	0.094	0.101	-0.103	0.295
		**b_MeanRichness**	**-0.072**	**0.011**	**-0.094**	**-0.052**
		b_Duration	-0.009	0.007	-0.022	0.005
		sd_SiteSubsite__Intercept	0.39	0.045	0.309	0.484
		phi	15.731	0.766	14.275	17.27
		lprior	-12.417	0.065	-12.545	-12.293
**39**	Losses FG - Graminoid	b_Intercept	-0.493	0.149	-0.779	-0.203
		b_zi_Intercept	-0.941	0.062	-1.064	-0.822
		b_GramDivided	0.153	0.12	-0.077	0.385
		b_LogPlotSize	0.093	0.101	-0.098	0.29
		**b_MeanRichness**	**-0.072**	**0.011**	**-0.093**	**-0.052**
		b_Duration	-0.008	0.007	-0.021	0.006
		sd_SiteSubsite__Intercept	0.392	0.045	0.307	0.484
		phi	15.764	0.766	14.271	17.309
		lprior	-12.418	0.065	-12.546	-12.29
**40**	Losses PCHG - Shrub	b_Intercept	-1.023	0.201	-1.419	-0.633
		b_zi_Intercept	-1.033	0.069	-1.172	-0.897
		**b_WarmQSlope**	**14.229**	**4.304**	**5.813**	**22.849**
		**b_PrecSlope**	**0.029**	**0.014**	**0.002**	**0.057**
		**b_ShrubSlope**	**0.05**	**0.014**	**0.022**	**0.078**
		b_LogPlotSize	0.157	0.099	-0.032	0.357
		**b_MeanRichness**	**-0.059**	**0.011**	**-0.08**	**-0.038**
		**b_Duration**	**-0.016**	**0.007**	**-0.03**	**-0.002**

		sd_SiteSubsite__Intercept	0.359	0.045	0.279	0.456
		phi	16.765	0.87	15.131	18.546
		lprior	-12.55	0.071	-12.69	-12.411
**41**	Losses PCHG - Forb	b_Intercept	-0.834	0.218	-1.266	-0.409
		b_zi_Intercept	-1.256	0.077	-1.409	-1.109
		**b_WarmQSlope**	**11.445**	**4.832**	**2.161**	**20.783**
		**b_PrecSlope**	**0.036**	**0.015**	**0.006**	**0.066**
		b_ForbSlope	-0.031	0.02	-0.071	0.007
		b_LogPlotSize	0.193	0.107	-0.015	0.404
		**b_MeanRichness**	**-0.068**	**0.011**	**-0.089**	**-0.046**
		b_Duration	-0.014	0.007	-0.028	0.0003
		sd_SiteSubsite__Intercept	0.387	0.048	0.3	0.488
		phi	15.75	0.848	14.139	17.423
		lprior	-12.589	0.077	-12.739	-12.434
**42**	Losses PCHG - Graminoid	b_Intercept	-0.897	0.194	-1.282	-0.516
		b_zi_Intercept	-0.965	0.063	-1.087	-0.843
		**b_WarmQSlope**	**12.857**	**4.314**	**4.533**	**21.357**
		**b_PrecSlope**	**0.03**	**0.014**	**0.003**	**0.058**
		**b_GraminoidSlope**	**-0.036**	**0.016**	**-0.067**	**-0.005**
		b_LogPlotSize	0.153	0.099	-0.036	0.35
		**b_MeanRichness**	**-0.073**	**0.01**	**-0.094**	**-0.053**
		b_Duration	-0.012	0.007	-0.025	0.001
		sd_SiteSubsite__Intercept	0.359	0.043	0.281	0.449
		phi	15.697	0.75	14.227	17.176
		lprior	-12.435	0.064	-12.56	-12.308
**43**	Gains GEO	b_Intercept	-1.448	0.627	-2.656	-0.187
		b_zi_Intercept	-1.275	0.068	-1.411	-1.145
		b_LAT	0.009	0.009	-0.009	0.026
		b_RegionGreenIceLand	0.342	0.124	0.101	0.584
		b_RegionNorthAmericaMEast	0.013	0.147	-0.279	0.298
		b_RegionNorthAmericaMWest	-0.016	0.082	-0.174	0.143
		b_LogPlotSize	0.03	0.083	-0.133	0.194
		**b_MeanRichness**	**-0.057**	**0.009**	**-0.076**	**-0.039**
		b_Duration	0.005	0.006	-0.007	0.017
		sd_SiteSubsite__Intercept	0.218	0.037	0.148	0.295
		phi	14.426	0.661	13.134	15.734
		lprior	-12.478	0.066	-12.606	-12.35
**44**	Gains CLIM	b_Intercept	-0.723	0.175	-1.062	-0.383
		b_zi_Intercept	-1.263	0.069	-1.4	-1.129
		b_MOISTUREMOIST	0.007	0.087	-0.168	0.176
		b_MOISTUREWET	0.062	0.119	-0.176	0.298
		**b_warmq**	**-0.034**	**0.016**	**-0.065**	**-0.003**
		b_prec	0.0002	0.0002	-0.0002	0.001
		b_Duration	0.009	0.006	-0.002	0.021
		b_LogPlotSize	-0.039	0.097	-0.233	0.153
		**b_MeanRichness**	**-0.057**	**0.009**	**-0.074**	**-0.04**
		sd_SiteSubsite__Intercept	0.208	0.037	0.14	0.284
		phi	14.641	0.682	13.332	16.001
		lprior	-12.491	0.067	-12.622	-12.363
**45**	Gains FG - Shrub	b_Intercept	-0.768	0.133	-1.03	-0.515
		b_zi_Intercept	-1.276	0.068	-1.409	-1.142
		b_ShrubDivided	-0.125	0.095	-0.312	0.065

		b_LogPlotSize	-0.067	0.076	-0.213	0.085
		**b_MeanRichness**	**-0.065**	**0.009**	**-0.082**	**-0.047**
		b_Duration	0.011	0.006	0.0002	0.022
		sd_SiteSubsite__Intercept	0.233	0.037	0.165	0.31
		phi	14.432	0.667	13.145	15.763
		lprior	-12.476	0.066	-12.606	-12.344
**46**	Gains FG - Forb	b_Intercept	-0.842	0.119	-1.068	-0.604
		b_zi_Intercept	-1.276	0.068	-1.407	-1.146
		b_ForbDivided	0.077	0.237	-0.388	0.541
		b_LogPlotSize	-0.071	0.076	-0.213	0.085
		**b_MeanRichness**	**-0.064**	**0.009**	**-0.082**	**-0.046**
		b_Duration	0.01	0.006	-0.001	0.021
		sd_SiteSubsite__Intercept	0.233	0.037	0.166	0.31
		phi	14.407	0.671	13.126	15.747
		lprior	-12.475	0.068	-12.609	-12.341
**47**	Gains FG - Graminoid	b_Intercept	-0.9	0.128	-1.148	-0.645
		b_zi_Intercept	-1.278	0.067	-1.409	-1.148
		b_GramDivided	0.131	0.101	-0.066	0.329
		b_LogPlotSize	-0.073	0.075	-0.214	0.082
		**b_MeanRichness**	**-0.063**	**0.009**	**-0.08**	**-0.046**
		b_Duration	0.011	0.006	-0.00006	0.023
		sd_SiteSubsite__Intercept	0.234	0.037	0.164	0.31
		phi	14.431	0.677	13.142	15.763
		lprior	-12.477	0.067	-12.607	-12.347
**48**	Gains PCHG - Shrub	b_Intercept	-1.113	0.162	-1.438	-0.799
		b_zi_Intercept	-1.456	0.079	-1.613	-1.306
		**b_WarmQSlope**	**7.599**	**3.399**	**1.032**	**14.337**
		b_PrecSlope	0.012	0.011	-0.009	0.034
		b_ShrubSlope	-0.019	0.014	-0.046	0.009
		b_LogPlotSize	-0.032	0.082	-0.189	0.132
		**b_MeanRichness**	**-0.059**	**0.009**	**-0.078**	**-0.04**
		b_Duration	0.007	0.006	-0.005	0.019
		sd_SiteSubsite__Intercept	0.253	0.038	0.183	0.332
		phi	14.956	0.737	13.566	16.503
		lprior	-12.631	0.077	-12.784	-12.481
**49**	Gains PCHG - Forb	b_Intercept	-1.182	0.174	-1.522	-0.837
		b_zi_Intercept	-1.64	0.088	-1.816	-1.469
		**b_WarmQSlope**	**8.831**	**3.582**	**1.894**	**16.114**
		b_PrecSlope	0.015	0.012	-0.008	0.038
		**b_ForbSlope**	**0.084**	**0.02**	**0.043**	**0.124**
		b_LogPlotSize	-0.024	0.081	-0.175	0.141
		**b_MeanRichness**	**-0.061**	**0.01**	**-0.08**	**-0.041**
		b_Duration	0.009	0.006	-0.003	0.021
		sd_SiteSubsite__Intercept	0.235	0.044	0.155	0.325
		phi	14.418	0.726	12.972	15.883
		lprior	-12.713	0.085	-12.882	-12.548
**50**	Gains PCHG - Graminoid	b_Intercept	-1.114	0.151	-1.408	-0.811
		b_zi_Intercept	-1.314	0.071	-1.455	-1.177
		**b_WarmQSlope**	**8.648**	**3.099**	**2.647**	**14.851**
		b_PrecSlope	0.01	0.01	-0.01	0.031
		b_GraminoidSlope	-0.027	0.015	-0.057	0.002
		b_LogPlotSize	-0.039	0.076	-0.186	0.119

		**b_MeanRichness**	**-0.065**	**0.009**	**-0.082**	**-0.047**
		b_Duration	0.008	0.006	-0.004	0.018
		sd_SiteSubsite__Intercept	0.221	0.037	0.154	0.297
		phi	14.424	0.667	13.171	15.747
		lprior	-12.505	0.067	-12.631	-12.374
**Temporal models**						
**51**	Richness change SUBS	b_Intercept	0.035	0.185	-0.323	0.405
		b_LAT	-0.001	0.003	-0.006	0.004
		b_RegionGreenIceLand	0.047	0.037	-0.026	0.12
		b_RegionNorthAmericaMEast	-0.015	0.044	-0.104	0.073
		b_RegionNorthAmericaMWest	0.0004	0.026	-0.051	0.052
		b_LogPlotSize	0.007	0.024	-0.038	0.054
		b_Duration	0.002	0.002	-0.001	0.006
		sd_SiteSubsite__Intercept	0.078	0.011	0.059	0.101
		sigma	0.162	0.003	0.156	0.169
		lprior	-4.369	0	-4.369	-4.368
**52**	Richness change PCHG - Shrub	b_Intercept	-0.024	0.047	-0.116	0.068
		b_MOISTUREMOIST	0.032	0.024	-0.017	0.08
		b_MOISTUREWET	0.051	0.037	-0.021	0.125
		b_WarmQSlope	-1.827	1.162	-4.109	0.467
		b_PrecSlope	0.001	0.003	-0.006	0.008
		**b_ShrubSlope**	**-0.011**	**0.004**	**-0.018**	**-0.004**
		b_LogPlotSize	-0.018	0.025	-0.067	0.031
		**b_Duration**	**0.004**	**0.002**	**0.0002**	**0.007**
		sd_SiteSubsite__Intercept	0.072	0.011	0.051	0.095
		sigma	0.157	0.004	0.15	0.164
		lprior	-4.368	0	-4.369	-4.368
**52b**	Richness change PCHG – with shrub categories interaction	b_Intercept	0.001	0.007	-0.014	0.016
		FGSlope	0.004	0.005	-0.006	0.014
		FuncGroupNonDwarfShrubCoverNew	0.002	0.006	-0.009	0.014
		**FGSlope: FuncGroupNonDwarfShrubCoverNew**	**-0.013**	**0.006**	**-0.025**	**-0.001**
		Sd_SiteSubsite__Intercept	0.04	0.006	0.02	0.05
		sigma	0.114	0.002	0.110	0.118
		lprior	-4.366	7.678	-4.367	-4.367
**53**	Richness change PCHG - Forb	b_Intercept	-0.082	0.054	-0.187	0.026
		b_MOISTUREMOIST	0.036	0.027	-0.017	0.089
		b_MOISTUREWET	0.024	0.038	-0.053	0.097
		b_WarmQSlope	-0.814	1.353	-3.503	1.885
		b_PrecSlope	0.002	0.004	-0.006	0.009
		**b_ForbSlope**	**0.028**	**0.005**	**0.018**	**0.039**
		b_LogPlotSize	-0.012	0.027	-0.066	0.042
		**b_Duration**	**0.004**	**0.002**	**0.001**	**0.008**
		sd_SiteSubsite__Intercept	0.079	0.012	0.057	0.105
		sigma	0.169	0.004	0.161	0.177
		lprior	-4.369	0	-4.37	-4.369
**54**	Richness change PCHG - Graminoid	b_Intercept	-0.04	0.043	-0.124	0.045
		b_MOISTUREMOIST	0.041	0.023	-0.005	0.087
		b_MOISTUREWET	0.024	0.031	-0.037	0.085
		b_WarmQSlope	-1.576	1.12	-3.78	0.648
		b_PrecSlope	0.001	0.003	-0.005	0.007
		b_GraminoidSlope	-0.005	0.004	-0.012	0.003

		b_LogPlotSize	-0.019	0.024	-0.067	0.029
		**b_Duration**	**0.004**	**0.002**	**0.001**	**0.007**
		sd_SiteSubsite__Intercept	0.067	0.011	0.047	0.089
		sigma	0.161	0.004	0.154	0.168
		lprior	-4.369	0	-4.369	-4.368
**55**	Evenness SUBS	b_Intercept	-0.023	0.018	-0.059	0.012
		b_LAT	0.0003	0.0003	-0.0002	0.001
		b_RegionGreenIceLand	-0.001	0.003	-0.008	0.006
		b_RegionNorthAmericaMEast	-0.007	0.004	-0.016	0.001
		b_RegionNorthAmericaMWest	0.001	0.002	-0.004	0.006
		b_MeanRichness	0.0003	0.0002	-0.00009	0.001
		b_Duration	-0.00005	0.0002	-0.0003	0.0003
		sd_SiteSubsite__Intercept	0.009	0.001	0.007	0.011
		sigma	0.012	0.0003	0.012	0.013
		lprior	-4.365	0	-4.365	-4.365
**56**	Evenness PCHG - Shrub	b_Intercept	0.002	0.005	-0.007	0.011
		b_MOISTUREMOIST	0.002	0.002	-0.003	0.006
		b_MOISTUREWET	0.011	0.004	0.004	0.018
		b_WarmQSlope	-0.109	0.112	-0.335	0.101
		b_PrecSlope	-0.0001	0.0003	-0.001	0.001
		**b_ShrubSlope**	**-0.002**	**0.0003**	**-0.003**	**-0.002**
		b_MeanRichness	0.00034	0.0002	-0.00002	0.001
		b_Duration	-0.0001	0.0002	-0.0004	0.0002
		sd_SiteSubsite__Intercept	0.008	0.001	0.006	0.01
		sigma	0.011	0.0003	0.01	0.011
		lprior	-4.365	0	-4.365	-4.365
**57**	Evenness PCHG - Forb	b_Intercept	0.001	0.005	-0.008	0.01
		b_MOISTUREMOIST	0.002	0.002	-0.003	0.006
		b_MOISTUREWET	0.009	0.003	0.003	0.015
		b_WarmQSlope	-0.1	0.107	-0.306	0.12
		b_PrecSlope	-0.0002	0.0003	-0.001	0.0004
		**b_ForbSlope**	**0.004**	**0.0003**	**0.003**	**0.004**
		**b_MeanRichness**	**0.0004**	**0.0002**	**0.00000**	**0.001**
		b_Duration	-0.0001	0.0001	-0.00037	0.0001
		sd_SiteSubsite__Intercept	0.007	0.001	0.006	0.009
		sigma	0.01	0.0003	0.01	0.011
		lprior	-4.365	0	-4.365	-4.365
**58**	Evenness PCHG - Graminoid	b_Intercept	0.001	0.005	-0.008	0.010
		b_MOISTUREMOIST	0.003	0.002	-0.002	0.008
		b_MOISTUREWET	0.009	0.003	0.003	0.015
		b_WarmQSlope	-0.101	0.12	-0.326	0.127
		b_PrecSlope	-0.0002	0.0003	-0.001	0.0004
		b_GraminoidSlope	0.0005	0.0003	-0.0001	0.001
		b_MeanRichness	0.0004	0.0002	-0.00001	0.001
		b_Duration	-0.0001	0.0002	-0.0004	0.0002
		sd_SiteSubsite__Intercept	0.008	0.001	0.006	0.01
		sigma	0.012	0	0.012	0.013
		lprior	-4.365	0	-4.365	-4.365
**59**	PCoA Jaccard Cartesian SUBS - Shrub	b_Intercept	-5.198	2.363	-9.823	-0.603
		b_RegionGreenIceLand	-1.503	0.492	-2.469	-0.527
		b_RegionNorthAmericaMEast	-1.679	0.626	-2.92	-0.435

		b_RegionNorthAmericaMWest	-0.055	0.404	-0.854	0.717
		b_MOISTUREMOIST	0.097	0.322	-0.528	0.736
		b_MOISTUREWET	0.987	0.484	-0.017	1.899
		b_SubsiteLatitude	0.011	0.035	-0.057	0.078
		**b_SubsiteTempChange**	**37.801**	**16.038**	**6.114**	**68.701**
		b_SubsitePrecChange	0.012	0.045	-0.078	0.098
		b_LogPlotSize	0.184	0.306	-0.412	0.795
		b_SubsiteRichness	0.018	0.048	-0.081	0.11
		b_SubsiteDuration	-0.022	0.022	-0.065	0.022
		b_SubsiteShrubChange	-0.095	0.111	-0.305	0.126
		phi	15.304	3.421	9.267	22.676
		lprior	-10.446	0.327	-11.055	-9.771
**60**	PCoA Jaccard Cartesian SUBS - Forb	b_Intercept	-3.408	3.336	-10.074	2.999
		b_RegionGreenIceLand	-0.839	0.666	-2.201	0.396
		b_RegionNorthAmericaMEast	-1.27	0.696	-2.668	0.045
		b_RegionNorthAmericaMWest	-1.188	0.411	-2.002	-0.388
		b_MOISTUREMOIST	0.47	0.359	-0.218	1.185
		b_MOISTUREWET	-0.816	0.609	-2.018	0.377
		b_SubsiteLatitude	0.014	0.045	-0.075	0.1
		b_SubsiteTempChange	3.505	21.279	-38.625	45.228
		b_SubsitePrecChange	-0.054	0.043	-0.143	0.031
		**b_LogPlotSize**	**1.244**	**0.393**	**0.482**	**2.022**
		b_SubsiteRichness	0.031	0.05	-0.065	0.13
		b_SubsiteDuration	-0.038	0.034	-0.103	0.032
		b_SubsiteForbChange	-0.151	0.143	-0.471	0.09
		phi	26.061	6.505	14.76	40.048
		lprior	-11.148	0.379	-11.85	-10.365
**61**	PCoA Jaccard Cartesian SUBS - Graminoid	b_Intercept	-5.043	2.177	-9.273	-0.73
		b_RegionGreenIceLand	-0.716	0.535	-1.782	0.321
		b_RegionNorthAmericaMEast	-0.558	0.594	-1.702	0.636
		b_RegionNorthAmericaMWest	-0.4	0.397	-1.161	0.376
		b_MOISTUREMOIST	-0.199	0.347	-0.852	0.497
		b_MOISTUREWET	-0.537	0.511	-1.541	0.446
		b_SubsiteLatitude	0.016	0.031	-0.046	0.077
		b_SubsiteTempChange	25.444	15.798	-6.66	55.221
		b_SubsitePrecChange	0.018	0.042	-0.067	0.097
		b_LogPlotSize	0.193	0.306	-0.385	0.806
		**b_SubsiteRichness**	**0.112**	**0.047**	**0.019**	**0.204**
		**b_SubsiteDuration**	**-0.066**	**0.024**	**-0.113**	**-0.018**
		b_SubsiteGraminoidChange	0.228	0.161	-0.104	0.535
		phi	13.502	2.982	8.239	19.903
		lprior	-10.307	0.322	-10.911	-9.643
**62**	PCoA Bray-Curtis Cartesian SUBS - Shrub	b_Intercept	-5.337	1.545	-8.435	-2.327
		b_RegionGreenIceLand	0.266	0.343	-0.431	0.921
		b_RegionNorthAmericaMEast	-0.067	0.423	-0.902	0.752
		b_RegionNorthAmericaMWest	-0.127	0.3	-0.715	0.471
		b_MOISTUREMOIST	0.037	0.24	-0.443	0.501
		b_MOISTUREWET	0.34	0.341	-0.365	0.977
		b_SubsiteLatitude	0.034	0.022	-0.011	0.078
		b_SubsiteTempChange	-14.701	13.577	-41.808	11.192
		b_SubsitePrecChange	0.013	0.037	-0.062	0.083
		b_LogPlotSize	-0.104	0.225	-0.531	0.369

		b_SubsiteRichness	0.02	0.039	-0.056	0.098
		b_SubsiteDuration	0	0.017	-0.033	0.034
		b_SubsiteShrubChange	0.005	0.088	-0.162	0.188
		phi	39.012	7.235	26.017	54.72
		lprior	-11.455	0.281	-12.009	-10.894
**63**	PCoA Bray-Curtis Cartesian SUBS - Forb	b_Intercept	-2.322	2.335	-7.041	2.111
		b_RegionGreenIceLand	-0.36	0.543	-1.475	0.679
		b_RegionNorthAmericaMEast	-0.156	0.487	-1.113	0.78
		b_RegionNorthAmericaMWest	-0.587	0.376	-1.311	0.158
		b_MOISTUREMOIST	0.232	0.277	-0.311	0.768
		b_MOISTUREWET	0.161	0.354	-0.566	0.826
		b_SubsiteLatitude	0.003	0.031	-0.059	0.064
		b_SubsiteTempChange	-27.158	17.613	-62.455	6.425
		b_SubsitePrecChange	-0.016	0.043	-0.103	0.069
		b_LogPlotSize	0.119	0.287	-0.411	0.7
		b_SubsiteRichness	0.015	0.04	-0.066	0.093
		b_SubsiteDuration	-0.005	0.021	-0.047	0.036
		b_SubsiteForbChange	0.01	0.09	-0.178	0.176
		phi	40.693	9.15	24.561	60.241
		lprior	-11.526	0.346	-12.181	-10.828
**64**	PCoA Bray-Curtis Cartesian SUBS - Graminoid	b_Intercept	-4.754	1.562	-7.875	-1.761
		b_RegionGreenIceLand	0.189	0.363	-0.535	0.886
		b_RegionNorthAmericaMEast	0.219	0.444	-0.656	1.075
		b_RegionNorthAmericaMWest	0.01	0.29	-0.553	0.589
		b_MOISTUREMOIST	-0.035	0.233	-0.482	0.427
		b_MOISTUREWET	0.095	0.285	-0.467	0.649
		b_SubsiteLatitude	0.027	0.022	-0.018	0.072
		b_SubsiteTempChange	-13.875	12.183	-38.861	9.102
		b_SubsitePrecChange	0.03	0.034	-0.04	0.096
		b_LogPlotSize	-0.109	0.219	-0.521	0.338
		b_SubsiteRichness	0.011	0.033	-0.055	0.078
		b_SubsiteDuration	-0.013	0.017	-0.046	0.019
		b_SubsiteGraminoidChange	-0.077	0.12	-0.314	0.154
		phi	38.981	6.963	26.525	53.764
		lprior	-11.44	0.272	-11.965	-10.892
**65**	PCoA Jaccard Centroid SUBS - Shrub	b_Intercept	-6.143	1.476	-9.068	-3.282
		b_RegionGreenIceLand	0.039	0.332	-0.627	0.682
		b_RegionNorthAmericaMEast	-0.172	0.384	-0.927	0.562
		b_RegionNorthAmericaMWest	-0.022	0.285	-0.599	0.529
		b_MOISTUREMOIST	-0.191	0.226	-0.628	0.26
		b_MOISTUREWET	0.724	0.291	0.122	1.28
		b_SubsiteLatitude	0.019	0.021	-0.022	0.059
		b_SubsiteTempChange	8.556	11.399	-14.046	30.601
		b_SubsitePrecChange	0.029	0.031	-0.033	0.087
		b_SubsiteShrubChange	-0.116	0.071	-0.249	0.032
		b_LogPlotSize	-0.034	0.228	-0.457	0.444
		b_SubsiteRichness	-0.026	0.034	-0.094	0.04
		b_SubsiteDuration	-0.016	0.016	-0.046	0.015
		phi	213.005	40.234	141.669	298.662
		lprior	-15.613	0.612	-16.853	-14.463
**66**		b_Intercept	-3.654	2.48	-8.618	0.994
		b_RegionGreenIceLand	-0.463	0.567	-1.635	0.617

	PCoA Jaccard Centroid SUBS - Forb	b_RegionNorthAmericaMEast	0.018	0.504	-0.968	0.981
		b_RegionNorthAmericaMWest	-0.595	0.347	-1.268	0.096
		b_MOISTUREMOIST	-0.006	0.284	-0.576	0.553
		b_MOISTUREWET	0.304	0.373	-0.436	1.018
		b_SubsiteLatitude	-0.008	0.034	-0.072	0.057
		b_SubsiteTempChange	7.164	17.567	-27.329	41.594
		b_SubsitePrecChange	0.004	0.036	-0.07	0.073
		b_SubsiteForbChange	0.01	0.094	-0.193	0.18
		b_LogPlotSize	0.093	0.272	-0.418	0.671
		b_SubsiteRichness	-0.024	0.039	-0.1	0.053
		b_SubsiteDuration	-0.035	0.022	-0.077	0.008
		phi	194.513	43.048	118.332	288.576
		lprior	-15.344	0.678	-16.744	-14.06
**67**	PCoA Jaccard Centroid SUBS - Graminoid	b_Intercept	-6.167	1.486	-9.1	-3.249
		b_RegionGreenIceLand	0.216	0.366	-0.514	0.927
		b_RegionNorthAmericaMEast	0.104	0.408	-0.699	0.917
		b_RegionNorthAmericaMWest	-0.302	0.284	-0.847	0.254
		b_MOISTUREMOIST	-0.353	0.238	-0.811	0.116
		b_MOISTUREWET	0.244	0.282	-0.323	0.794
		b_SubsiteLatitude	0.026	0.021	-0.016	0.066
		b_SubsiteTempChange	3.082	12.35	-21.915	26.74
		b_SubsitePrecChange	0.036	0.029	-0.021	0.092
		b_SubsiteGraminoidChange	0.166	0.106	-0.048	0.365
		b_LogPlotSize	-0.005	0.233	-0.441	0.469
		b_SubsiteRichness	0.002	0.033	-0.066	0.069
		**b_SubsiteDuration**	**-0.045**	**0.018**	**-0.079**	**-0.01**
		phi	198.172	35.897	133.607	273.893
		lprior	-15.417	0.561	-16.553	-14.341
**68**	PCoA Bray-Curtis Centroid SUBS - Shrub	b_Intercept	-5.771	1.738	-9.213	-2.464
		b_RegionGreenIceLand	-0.459	0.392	-1.256	0.288
		b_RegionNorthAmericaMEast	-0.655	0.428	-1.495	0.171
		b_RegionNorthAmericaMWest	-0.2	0.288	-0.763	0.374
		b_MOISTUREMOIST	0.383	0.239	-0.087	0.868
		b_MOISTUREWET	0.724	0.341	0.024	1.361
		b_SubsiteLatitude	0.022	0.025	-0.027	0.07
		b_SubsiteTempChange	-6.215	14.332	-34.66	21.525
		b_SubsitePrecChange	0.011	0.032	-0.053	0.073
		b_SubsiteShrubChange	0.033	0.097	-0.147	0.231
		b_LogPlotSize	-0.033	0.245	-0.496	0.469
		b_SubsiteRichness	-0.024	0.036	-0.095	0.047
		b_SubsiteDuration	0.013	0.018	-0.023	0.048
		phi	102.964	19.509	68.398	143.744
		lprior	-13.59	0.412	-14.388	-12.806
**69**	PCoA Bray-Curtis Centroid SUBS - Forb	b_Intercept	-8.32	3.018	-14.413	-2.611
		b_RegionGreenIceLand	-0.46	0.585	-1.651	0.653
		b_RegionNorthAmericaMEast	-0.437	0.548	-1.542	0.619
		b_RegionNorthAmericaMWest	0.058	0.411	-0.713	0.907
		b_MOISTUREMOIST	0.277	0.307	-0.31	0.9
		b_MOISTUREWET	0.353	0.369	-0.375	1.071
		b_SubsiteLatitude	0.041	0.039	-0.037	0.119
		b_SubsiteTempChange	9.93	16.756	-22.711	42.726
		b_SubsitePrecChange	0.058	0.043	-0.028	0.143

		b_SubsiteForbChange	0.028	0.111	-0.218	0.216
		b_LogPlotSize	-0.095	0.318	-0.681	0.563
		b_SubsiteRichness	0.009	0.041	-0.072	0.089
		b_SubsiteDuration	0.006	0.023	-0.042	0.05
		phi	109.169	25.218	65.793	164.58
		lprior	-13.788	0.52	-14.831	-12.787
**70**	PCoA Bray-Curtis Centroid SUBS - Graminoid	b_Intercept	-4.72	1.719	-8.049	-1.296
		b_RegionGreenIceLand	-0.545	0.414	-1.372	0.258
		b_RegionNorthAmericaMEast	-0.159	0.449	-1.044	0.712
		b_RegionNorthAmericaMWest	-0.305	0.286	-0.87	0.257
		b_MOISTUREMOIST	0.522	0.263	0.009	1.047
		b_MOISTUREWET	0.096	0.329	-0.545	0.738
		b_SubsiteLatitude	0.005	0.025	-0.046	0.053
		b_SubsiteTempChange	-1.642	13.168	-28.256	22.955
		b_SubsitePrecChange	0.028	0.03	-0.032	0.085
		b_SubsiteGraminoidChange	-0.103	0.119	-0.342	0.123
		b_LogPlotSize	0.149	0.253	-0.32	0.67
		b_SubsiteRichness	-0.024	0.035	-0.091	0.042
		b_SubsiteDuration	0.007	0.018	-0.027	0.042
		phi	104.578	19.384	69.63	146.053
		lprior	-13.645	0.408	-14.459	-12.853

Supplementary Table 4. Comparison of model outputs for a selection of models that were fitted both as univariate and as multivariate models (**Supplementary Table 2**). We consider a slope from a univariate model to be significant when the 95% credible intervals did not overlap zero. We consider a slope from the multivariate model to be significant when the 95% or the 97.5% credible intervals did not overlap zero (**Supplementary Table 2**). The richness change ~ temperature change model was run at the subsite-level by extracting subsite-level slopes from the richness over time hierarchical model (**Supplementary Table 1**). Positive and negative significant relationships are indicated by the symbols '+' and '-', respectively, while 'ns' indicates a non-significant relationship.

Relationship	Univariate model	Multivariate model
Richness change ~ temperature change	ns	ns
Jaccard ~ temperature	-	-
Jaccard ~ temperature change	+	+
Bray-Curtis ~ temperature	+	ns
Bray-Curtis ~ temperature change	ns	-
Species losses ~ temperature	-	-
Species losses ~ precipitation	ns	+
Species losses ~ shrub change	+	+
Species losses ~ temperature change	+	+
Species gains ~ temperature	-	-
Species gains ~ shrub change	ns	ns
Species gains ~ temperature change	+	+

Supplementary Table 5. Structure and summary results of the model behind **Extended Data Figure 7a**. We report slopes and 95% credible intervals. Only plots where shrub cover had increased over time are included.

Model structure	Sample size	Summary
Richness change ~ Shrub change over time * Start shrub cover + (1|SiteSubsite)	528	Shrub change over time: -0.02 (CI = -0.047 to 0.0026)<br>Start shrub cover: -0.0006 (CI = -0.0014 to 0.0002)<br>Shrub change over time * Start shrub cover: -0.0003 (CI = -0.0009 to 0.0002)

Supplementary Table 6. Top 10 species in each trajectory, ordered by the number of times a species has been identified as a persisting, becoming lost or gained across all plots. Percentages per trend are calculated relative to the overall number of times a species has been recorded across plots (considering unique trajectories only as opposed to multiple timepoints). FG = functional group.

	Persisters				Losses				Gains			
	**Species**	**FG**	**#**	**%**	**Species**	**FG**	**#**	**%**	**Species**	**FG**	**#**	**%**
**1**	*Vaccinium vitis-idaea*	Shrub	471	87.2	*Luzula confusa*	Graminoid	57	26.3	*Carex bigelowii*	Graminoid	59	11.5
**2**	*Carex bigelowii*	Graminoid	403	78.4	*Carex bigelowii*	Graminoid	52	10.1	*Betula nana*	Shrub	55	15.2
**3**	*Carex aquatilis*	Graminoid	286	92.9	*Salix herbacea*	Shrub	46	23.2	*Poa arctica*	Graminoid	53	21.0
**4**	*Betula nana*	Shrub	276	76.2	*Poa arctica*	Graminoid	42	16.6	*Luzula confusa*	Graminoid	51	23.5
**5**	*Cassiope tetragona*	Shrub	271	88.3	*Carex lachenalii*	Graminoid	38	82.6	*Arctagrostis latifolia*	Graminoid	47	25.0
**6**	*Salix arctica*	Shrub	233	81.8	*Stellaria longipes*	Forb	37	30.1	*Vaccinium vitis-idaea*	Shrub	47	8.7
**7**	*Eriophorum vaginatum*	Graminoid	189	85.1	*Saxifraga cernua*	Forb	36	25.7	*Phyllodoce caerulea*	Shrub	47	39.8
**8**	*Empetrum nigrum*	Shrub	181	74.2	*Armeria maritima*	Forb	36	36.0	*Petasites frigidus*	Forb	45	26.6
**9**	*Empetrum hermaphroditum*	Shrub	160	78.4	*Bistorta vivipara*	Forb	35	22.2	*Vaccinium myrtillus*	Shrub	41	20.6
**10**	*Dupontia fisheri*	Graminoid	159	74.3	*Arctagrostis latifolia*	Graminoid	34	18.1	*Cassiope hypnoides*	Shrub	41	40.6

Supplementary Table 7. Structure and summary results of the *post hoc* analyses behind **Extended Data Figure 9**. We report slopes and 95% credible intervals. Bold indicates terms whose credible intervals do not overlap zero.

**Model structure**	**Sample size**	**Summary**
Shrub cover increases ~ Latitude + (1|Subsite)	503	Latitude: -0.004 (CI = -0.04 to 0.03)
Shrub cover ~ Temperature mean of past 5 years * Shrub type + (Temperature mean of past 5 years | Subsite/Plot)	6,715	**Temperature mean of past 5 years: -0.12 (CI: -0.24 to -0.004)**<br>**Erect Shrubs: 1.40 (CI: 1.35 to 1.44)**<br>**Temperature mean of past 5 years * Erect Shrubs: 0.20 (CI: 0.08 to 0.32)**
Shrub cover change ~ Temperature change * Shrub type + (1|Subsite)	665	Erect shrubs: 1.35 (-0.88 to 3.66)<br>Temperature change: 33.83 (CI = -16.61 to 86.41)<br>Temperature change * Erect shrubs: -18.26 (-78.88 to 41.33)

Supplementary Table 8. Number and percentage of plots with different trends for each of the three functional groups. Increasing, stable and decreasing trends are defined by the change in cover values per functional group per plot. Never present indicates those functional groups that were not present in a plot either the start or the end monitoring time point.

Trend	Shrub	Forb	Graminoid
Decrease	556 (43.9%)	490 (38.7%)	599 (47.3%)
Increase	527 (41.6%)	483 (38.1%)	588 (46.4%)
Stable	7 (0.5%)	2 (0.2%)	46 (3.6%)
Never present	176(13.9%)	291 (22.9%)	33 (2.6%)

Supplementary Table 9. Structure and summary results of the functional group change over time models. CI are the 95% credible intervals. The response variables are the slopes of change per functional group, as calculated in a linear model per plot (as cover in functional group ~ year). All estimates and credible intervals overlapped zero.

Model structure	Sample size	Summary
Shrub change over time ~ 1 + (1|SiteSubsite)	1,091	0.05 (CI = -0.21 to 0.3)
Forb change over time ~ 1 + (1|SiteSubsite)	995	0.09 (CI = -0.12 to 0.31)
Graminoid change over time ~ 1 + (1|SiteSubsite)	1,240	-0.15 (CI = -0.36 to 0.05)
