# Supplementary material for: Plant diversity dynamics over space and time in a warming Arctic
Source: Nature. 2025 Apr 30;642(8068):653–61. doi: 10.1038/s41586-025-08946-8 (PMC12176628; doi:10.1038/s41586-025-08946-8)
Supplement: Supplementary file 2 — Reporting Summary [file 41586_2025_8946_MOESM2_ESM.pdf]

nature portfolio

Corresponding author(s): Mariana García Criado

Last updated by author(s): Feb 11, 2025

Reporting Summary

Nature Portfolio wishes to improve the reproducibility of the work that we publish. This form provides structure for consistency and transparency in reporting. For further information on Nature Portfolio policies, see our Editorial Policies and the Editorial Policy Checklist.

Statistics

For all statistical analyses, confirm that the following items are present in the figure legend, table legend, main text, or Methods section.

n/a	Confirmed	
☐	☒	The exact sample size (*n*) for each experimental group/condition, given as a discrete number and unit of measurement
☐	☒	A statement on whether measurements were taken from distinct samples or whether the same sample was measured repeatedly
☐	☒	The statistical test(s) used AND whether they are one- or two-sided<br>*Only common tests should be described solely by name; describe more complex techniques in the Methods section.*
☐	☒	A description of all covariates tested
☐	☒	A description of any assumptions or corrections, such as tests of normality and adjustment for multiple comparisons
☐	☒	A full description of the statistical parameters including central tendency (e.g. means) or other basic estimates (e.g. regression coefficient) AND variation (e.g. standard deviation) or associated estimates of uncertainty (e.g. confidence intervals)
☐	☒	For null hypothesis testing, the test statistic (e.g. *F*, *t*, *r*) with confidence intervals, effect sizes, degrees of freedom and *P* value noted<br>*Give P values as exact values whenever suitable.*
☐	☒	For Bayesian analysis, information on the choice of priors and Markov chain Monte Carlo settings
☐	☒	For hierarchical and complex designs, identification of the appropriate level for tests and full reporting of outcomes
☒	☐	Estimates of effect sizes (e.g. Cohen's *d*, Pearson's *r*), indicating how they were calculated

Our web collection on statistics for biologists contains articles on many of the points above.

Software and code

Policy information about availability of computer code

Data collection	No specific software was used to collect data. We used the software and programming language R version 4.1.0 (R Core Team, 2022) to produce the input data file of plant composition.
Data analysis	We used the software and programming language R version 4.1.0 (R Core Team, 2022). Bayesian hierarchical models were fitted using the ‘brms’ package v2.17 in R (Bürkner, 2017). Principal Coordinate Analyses were carried out with the ‘vegan’ v2.6-2 (Oksanen et al. 2020) and ‘ape’ v5.6-2 (Paradis & Schliep, 2018) packages in R. Polar projection maps were created with the 'ggOceansMapsData' package v1.454 (Vihtakari 2024). The R code to generate the figures and analyses of this manuscript is accessible at https://doi.org/10.5281/zenodo.14884498

For manuscripts utilizing custom algorithms or software that are central to the research but not yet described in published literature, software must be made available to editors and reviewers. We strongly encourage code deposition in a community repository (e.g. GitHub). See the Nature Portfolio guidelines for submitting code & software for further information.

Data

Policy information about availability of data

All manuscripts must include a data availability statement. This statement should provide the following information, where applicable:

- Accession codes, unique identifiers, or web links for publicly available datasets
- A description of any restrictions on data availability
- For clinical datasets or third party data, please ensure that the statement adheres to our policy

Plant composition data is available at https://doi.org/10.5281/zenodo.14884498. Climate data from CHELSA can be accessed at https://chelsa-climate.org/ and snow data is available at https://springernature.figshare.com/collections/ARCLIM_bioclimatic_indices_for_the_terrestrial_Arctic/6216368

Research involving human participants, their data, or biological material

Policy information about studies with human participants or human data. See also policy information about sex, gender (identity/presentation), and sexual orientation and race, ethnicity and racism.

Reporting on sex and gender	Not applicable.
Reporting on race, ethnicity, or other socially relevant groupings	Not applicable.
Population characteristics	Not applicable.
Recruitment	Not applicable.
Ethics oversight	Not applicable.

Note that full information on the approval of the study protocol must also be provided in the manuscript.

Field-specific reporting

Please select the one below that is the best fit for your research. If you are not sure, read the appropriate sections before making your selection.

☐ Life sciences ☐ Behavioural & social sciences ☒ Ecological, evolutionary & environmental sciences

For a reference copy of the document with all sections, see nature.com/documents/nr-reporting-summary-flat.pdf

Ecological, evolutionary & environmental sciences study design

All studies must disclose on these points even when the disclosure is negative.

Study description	This study quantifies local-scale plant species richness and composition and its change over time across plots in the Arctic, and identifies the geographic, climatic and biotic drivers behind these changes. The majority of models are Bayesian hierarchical models with a subsite random effect. Data families were chosen depending on the structure of the response variable and include Gaussian, negative binomial, beta, zero-inflated-beta and zero-one-inflated beta. Sample size was dependent on the response variable, and it is specified throughout the manuscript.
Research sample	We used a compilation of 42,234 records of 490 vascular plant species from 2,174 plots at 155 subsites within 45 study areas across the Arctic. Out of the 2,174 plots, 787 plots (36.2%) had only been surveyed once (and thus were only included in spatial analyses) and 1,266 (58.2%) plots were surveyed more than once and over a minimum period of five years (and thus were used for both spatial and temporal analyses).
Sampling strategy	We retained records that complied with our criteria (i.e., plots equal or smaller to 1m2, north of 60 degrees latitude, consistent surveying methods over time, only vascular plants, plots with <10% morphospecies). We used all available records per plot in order to appropriately capture local-scale plant diversity patterns and trends over time.
Data collection	Anne Bjorkman, Mariana García Criado and Sarah Elmendorf cleaned and curated the ITEX+ database.
Timing and spatial scale	The data in this manuscript span the period 1981-2022. Plots were monitored over different periods, with a mean study duration of 8 years (range = 1 to 28), a mean of 3 monitoring time points per plot (range = 1 to 11) and a mean time between surveys of 5 years (range = 1 to 26). Our dataset contains records from Europe, Greenland and North America north of 60 degrees latitude. Our database comprises 2,174 plots in 155 subsites within 45 study areas across the Arctic. Plots are equal or smaller to 1m2.
Data exclusions	Plant records from the ITEX+ database were excluded in the following instances: 1) records were obvious duplicates, 2) plots that were under 60 degrees latitude, 3) plots that had inconsistent surveying methods and/or plot sizes over time, 4) plots that were greater than 1m2, 5) plots that had >10% of morphospecies, and 6) records that were not vascular plants (i.e., non-vascular plants

and non-biotic records). For temporal analyses, plots were excluded when they had been surveyed less than twice and over a shorter time period than five years.

Reproducibility	All code and data are publicly available at https://doi.org/10.5281/zenodo.14884498
Randomization	Randomization was not applicable to this study as we required all available relevant records in order to capture local-scale plant diversity patterns and trends over time.
Blinding	Blinding was not applicable to this study as no participants were involved and no comparison was made between control and experimental studies.

Did the study involve field work? ☐ Yes ☒ No

Reporting for specific materials, systems and methods

We require information from authors about some types of materials, experimental systems and methods used in many studies. Here, indicate whether each material, system or method listed is relevant to your study. If you are not sure if a list item applies to your research, read the appropriate section before selecting a response.

Materials & experimental systems

n/a	Involved in the study	
☒	☐	Antibodies
☒	☐	Eukaryotic cell lines
☒	☐	Palaeontology and archaeology
☒	☐	Animals and other organisms
☒	☐	Clinical data
☒	☐	Dual use research of concern
☒	☐	Plants

Methods

n/a	Involved in the study	
☒	☐	ChIP-seq
☒	☐	Flow cytometry
☒	☐	MRI-based neuroimaging
